# Self-Healing Imidazole-Cured Epoxy Using Microencapsulated Epoxy-Amine Chemistry

**DOI:** 10.3390/polym17172391

**Published:** 2025-09-01

**Authors:** Zhihui Li, Gang Du, Sen Yang, Xuerong Lu, Fuli Zheng, Bin Hao, Peng Zhan, Guangmao Li, He Zhang

**Affiliations:** 1 Guangzhou Power Supply Bureau Electric Power Research Institute, Guangzhou 510645, Chinalgm1987@163.com (G.L.); 2 Guangzhou HKUST Fok Ying Tung Research Institute, Guangzhou 511462, China

**Keywords:** self-healing, epoxy, amine, microcapsule, imidazole

## Abstract

Epoxy resins used in reactors are prone to cracking and failure due to mechanical vibration, thermal stress, and ultraviolet radiation. Improving their resistance to damage is important to extend the service life of reactors. This investigation develops a self-healing imidazole-cured epoxy resin for reactors using epoxy microcapsules and amine microcapsules prepared by electrospraying-interfacial polymerization (ES-IP) microencapsulation technique. Firstly, this investigation studies the feasibility of using double nozzles for simultaneous spraying to improve the preparation of small-sized microcapsules. After successful synthesis, the healing performance of self-healing imidazole-cured epoxy based on the microencapsulated epoxy-amine chemistry was studied, focusing on the influence of the ratio, concentration, and size of the two microcapsules on the healing efficiency, and further exploring the thermal stability of the self-healing performance. The addition of microcapsules to the mechanical properties was also investigated. Results show that the double-nozzle technique can prepare microcapsules with controllable sizes (20~200 μm). The self-healing imidazole-cured epoxy exhibits high self-healing performance, reaching 100% at the optimal ratio with 10.0 wt% 50~100 μm microcapsules. Although the added microcapsules reduce the tensile strength of the material, they improve its high-temperature aging resistance. The above investigation is significant for developing self-healing fiber-reinforced epoxy-based composite materials for reactors.

## 1. Introduction

Dry-type air-core reactors, as key equipment in power systems, are widely used in current limiting, filtering, and reactive power compensation due to their advantages of light weight, low noise, high mechanical strength, and good tolerance. However, due to the long-term comprehensive effects of mechanical stress, thermal stress, electrical stress, and ultraviolet radiation, the epoxy resin composite encapsulation outside the reactor is prone to aging and cracking, which can lead to the intrusion of moisture and humidity into the reactor, seriously threatening the safe operation of the reactor, and even causing major accidents such as inter-turn discharge and fire [1,2,3,4]. According to statistics from China Southern Power Grid, the failure rate of dry-type air-core reactors is much higher than that of other power equipment, such as transformers and circuit breakers. Solving the cracking problem of reactor encapsulation materials is imminent.

At present, the traditional repair methods for the cracking problem of reactor encapsulation mainly include local filling and overall filling. Local filling usually uses room-temperature curable epoxy resins to inspect and repair microcracks on the surface of the encapsulation. Overall, filling is to re-spray insulating paint on the surface of the reactor to enhance the overall insulation and mechanical properties. However, these traditional methods have certain limitations. Local repair relies on the visibility of cracks, making it difficult to detect and repair early or subtle cracks. Overall, filling may have the problem of uneven paint filling. More importantly, both methods require the equipment to be powered off, which increases maintenance costs and time.

In recent years, self-healing materials, as an emerging technology, have provided new ideas for solving the above problems. Microcapsule-based self-healing materials microencapsulate healants in microcapsules. When the material cracks, the microcapsules rupture and release the healants, thereby achieving autonomous healing of the cracks. At present, microcapsule-based self-healing materials have made some progress in the repair of electrical tree damage in epoxy resins [5,6,7]. He et al. [6] has shown that microcapsules synthesized by the emulsion method can effectively repair electrical tree damage channels and increase the resistivity. Sima et al. [8] has shown that microwave/magnetic field dual-response self-healing microcapsules prepared by the emulsion method can repair electrical trees and cracks in the epoxy resin matrix, and have little impact on the electrical properties of the matrix material.

However, applying existing microcapsule self-healing technology to reactor encapsulation repair still faces some challenges. First, the cracking mechanism of reactor encapsulation is fundamentally different from internal electrical tree breakdown damage. The cracking of reactor encapsulation is mainly due to material aging caused by external environmental factors, while electrical tree damage is caused by internal electric field concentration breakdown. Second, existing research pays little attention to the impact of microcapsules on the mechanical properties of materials. As a fiber-reinforced composite, the mechanical properties of reactor encapsulation are crucial. If the addition of microcapsules leads to a significant decrease in the mechanical properties of the material, it will seriously affect the service life of the reactor. In addition, the microcapsules used in existing research have a large particle size, which may be difficult to apply to fiber-reinforced epoxy resin composite materials. Attributed to the limited space for resin and microcapsules in composite materials, the adopted microcapsules should be as small as possible [9,10,11].

Recently, based on the principle of directly encapsulating non-equilibrium droplets, Zhang et al. developed microencapsulation techniques via integrating microfluidic T-junction and interfacial polymerization (T-IP), electrospraying and interfacial polymerization (ES-IP), etc., and successfully prepared epoxy microcapsules and amine microcapsules with excellent comprehensive properties [12,13]. Compared with other microencapsulation techniques using in situ polymerization or interfacial polymerization in emulsions [14,15], the microcapsules prepared by this technique have low impurity content, high core fraction, controllable and adjustable size with relatively small dispersity, and good long-term and thermal stability [12,13]. Based on the synthesized high-quality epoxy microcapsules and amine microcapsules, they further studied the self-healing amine-cured epoxy resin at low temperatures based on the microencapsulated epoxy-amine chemistry [16,17]. The effects of the microcapsule ratio, microcapsule concentration, microcapsule size, and heat treatment conditions on the healing performance of this self-healing system. It shows that the self-healing system based on the microencapsulated epoxy-amine chemistry by the ES-IP microencapsulation technique has the advantages of high healing efficiency, relatively fast healing speed, good long-term stability and thermal stability, and is a self-healing system with potential practical applications.

In order to solve the above problem for the reactor, this investigation proposes a self-healing imidazole-cured epoxy resin based on microencapsulated epoxy-amine chemistry. This investigation uses the ES-IP microencapsulation technique to prepare epoxy microcapsules and amine microcapsules [12,18,19], and adds them to imidazole-cured epoxy to develop a new type of self-healing material. Compared with traditional self-healing systems, this system has the following advantages: (1) It can achieve autonomous healing of cracks in reactor encapsulation without power outage; (2) It can effectively solve the problem that traditional repair methods cannot repair early or subtle cracks; (3) By optimizing the type, ratio, and particle size of the microcapsules, the mechanical properties of the material can be taken into account while ensuring self-healing performance. This study aims to lay a theoretical foundation and technical support for the development of self-healing fiber-reinforced epoxy-based composite materials for reactor encapsulation.

## 2. Experiments Session

### 2.1. Materials

Decahydronaphthalene, *n*-hexadecane, cyclohexane, diethylenetriamine, diethylene diamine (DABCO), tetraethylenepentamine (TEPA), *n*-butyl glycidyl ether (BGE), bisphenol F diglycidyl ether (BFDGE), and sodium dodecyl sulfate (SDS) were purchased from McLean Biochemical Reagent Co., Ltd. (Shaihai, China). 4,4′-Dicyclohexylmethane diisocyanate (HMDI) was purchased from Yantai Wanhua Co. (Yantai, China). Polyetheramine JEFFAMINE T403 was provided by Huntsman (Salt Lake City, UT, USA). Surfactant Arlacel P135 was purchased from Croda International Plc. (Goole, UK). Epoxy resin for reactors and imidazole curing agent were self-developed and provided by Shunte Electric Equipment Co., Ltd. (Shunde, China). Epoxy resin Epolam 5015 and curing agent Hardener 5014 were purchased from Axson Technologies (Paris, France). All reagents were used directly after purchase.

### 2.2. Fabrication of Epoxy Microcapsules and Amine Microcapsules

**Figure 1:**
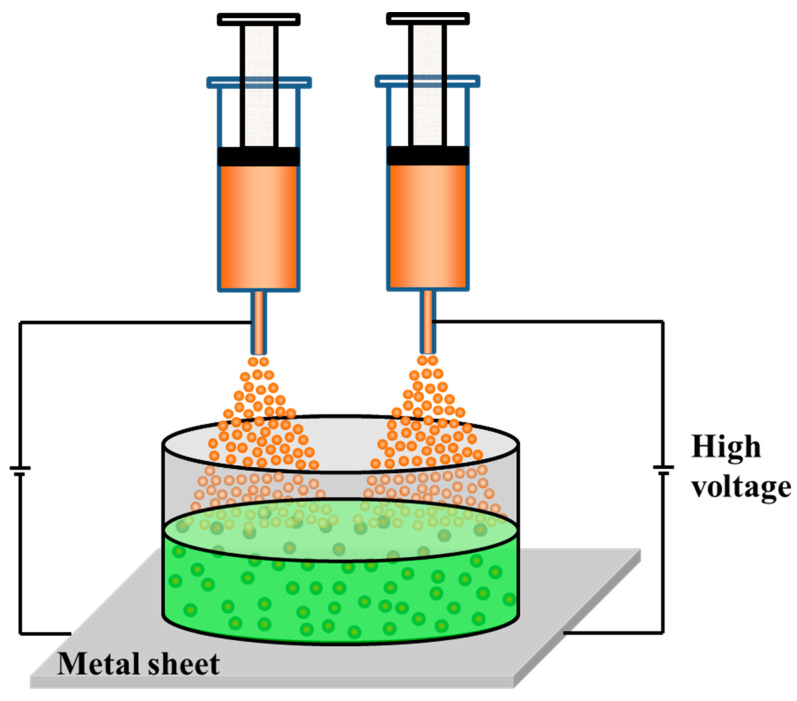
ES-IP microencapsulation technique using double nozzles.

Epoxy microcapsules and amine microcapsules were prepared using the ES-IP microencapsulation technique based on direct microencapsulation of non-equilibrium microdroplets developed by Zhang et al. [12]. To improve the preparation efficiency of small-sized microcapsules, this investigation adopts double nozzles for simultaneous spraying instead of single nozzles, as shown in Figure 1. Briefly, when preparing epoxy microcapsules, the epoxy core liquid containing 5.0 wt% HMDI, BFDGE diluted using 10.0 wt% BGE, is fed from the nozzle using a syringe pump at a certain feeding rate, and then atomized under electrostatic action to form microdroplets, which fall into a reaction solution containing 100 mL deionized water, 7.2 g shell-forming monomer diethylenetriamine, and 0.01 g surfactant SDS to form primary epoxy microcapsules. After electrostatic spraying was completed, the mixture was reacted at 90 °C for 10 h under mechanical stirring (DLAB; OS20-Pro) at 350 rpm to form the robust epoxy microcapsules. Finally, they were washed, separated with deionized water, and dried at room temperature (25 °C) to obtain the final epoxy microcapsules.

When preparing amine microcapsules, the amine core liquid, a mixture containing 85.0 wt% JEFFAMINE T403 and 15.0 wt% TEPA, is fed from the nozzle using a syringe pump (Leadfluid TYD02-02, Baoding, China) at a certain rate, and then atomized under electrostatic action to form microdroplets, which fall into a reaction solution containing 75 mL decahydronaphthalene, 75 mL *n*-hexadecane, 3.0 g surfactant Arlacel P135, 1.5 g accelerator DABCO, and 18.0 g shell-forming monomer HMDI to form primary amine microcapsules. After electrostatic spraying was completed, the mixture was reacted at 80 °C for 3 h under mechanical stirring (DLAB OS20-Pro, Beijing, China) at 350 rpm to form the robust amine microcapsules. Finally, they were washed, separated with cyclohexane, and dried at room temperature to obtain the final amine microcapsules.

To prepare epoxy microcapsules and amine microcapsules with different sizes, this investigation adopted different parameter combinations of feeding rate and spraying voltage, as shown in Table 1. The microencapsulation efficiency and the core fraction of the synthesized microcapsules were also listed in the table when the double-nozzle technique was adopted.

### 2.3. Formulation and Characterization of Self-Healing Epoxy

**Figure 2:**
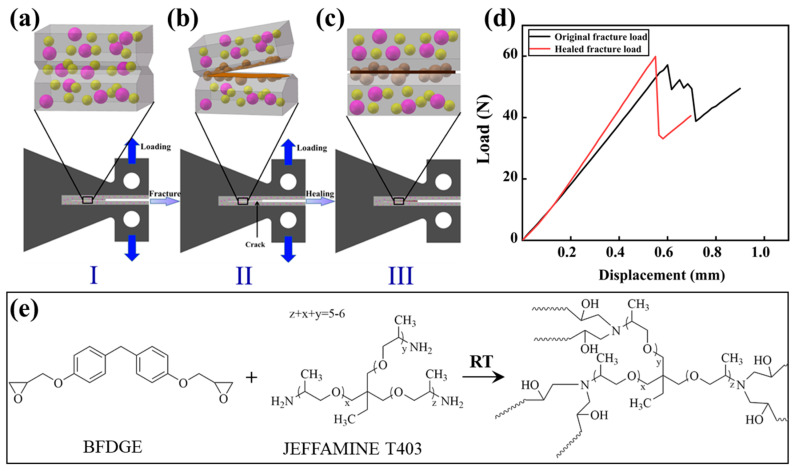
(**a**–**c**) Self-healing performance characterization for the formulated self-healing epoxy using TDCB specimen, and (**d**) typical force-displacement curves of the original fracture and healed fracture for the self-healing epoxy. (**e**) Curing reaction between the released epoxy monomer BFDGE and amine hardener JEFFAMINE T403, respectively, from the epoxy microcapsule and amine microcapsule.

As shown in Figure 2, the healing efficiency of the self-healing imidazole-cured epoxy based on mode I fracture toughness was tested using short-grove tapered double cantilever beam (TDCB) specimens (Figure 2a). First, Epolam 5015 and Hardener 5014 (100:34) were used to prepare the outer frame of the TDCB specimen. Due to the density difference, epoxy microcapsules and amine microcapsules are prone to floating and sinking in the epoxy adhesive, resulting in uneven distribution of microcapsules, which affects the self-healing performance. Therefore, after the microcapsules were mixed into the epoxy/imidazole adhesive (100:13), the mixture is pre-cured with rotation at 60 °C for 2 h to increase the viscosity of the mixture to avoid microcapsule floating/sinking before pouring, and finally cured under the recommended procedure of imidazole-cured epoxy resin (100 °C—2 h + 120 °C—4 h + 140 °C—5 h).

During the test, a crack of 8–10 mm long was pre-made on the self-healing sample, and then the universal testing machine (Instron 5567, Boston, MA, USA) was used to load it at a speed of 1 mm/min to fracture it (Figure 2b), and the original force-displacement curve of the self-healing sample was obtained (Figure 2d). The fractured sample was healed at room temperature (25 °C) for 48 h. During the healing process, the released epoxy BFDGE from the epoxy microcapsule was cured by the released amine hardener JEFFAMINE T403 from the amine microcapsule, as schematically shown in Figure 2e. And then the repaired sample was fractured again using the same equipment and parameters (Figure 2c), and the healing force-displacement curve of the self-healing sample was obtained (Figure 2d). According to the sample configuration, the self-healing efficiency can be defined as [20]:

(1) $$\eta =100\%\cdot \frac{K_{IC}^{Healed}}{K_{IC}^{Original}}=100\%\cdot \frac{P_{Average}^{Healed}}{P_{Average}^{Original}}$$

where $K_{IC}^{Original}$, $K_{IC}^{Healed}$, $P_{Average}^{Original}$, and $P_{Average}^{Healed}$ are the original mode I fracture toughness, the healed mode I fracture toughness, the averaged peak load of the original force-displacement curve, and the averaged peak load of the healed force-displacement curve, respectively. Table 2 shows the microcapsule parameters used in this study for the self-healing samples.

### 2.4. Mechanical Test

The tensile test of the self-healing samples was carried out according to ASTM D638. The loading rate of the samples was 1.0 mm/min. Table 3 lists the sample parameters for the tensile test. To reduce experimental errors, each sample was tested with six parallel replicates to obtain the average value and the standard deviation.

### 2.5. Other Characterizations

The morphology of the two microcapsules and the fracture surface of the epoxies were observed using a scanning electron microscope (FEI QUANTA FEG 250, Hillsboro, RE, USA). The microcapsule size was measured using Mastersizer 2000 (Malvern Panalytical, Malvern, UK). The size distribution of the epoxy microcapsules and amine microcapsules of 50~100 μm were given in Figure S1 in the Supplementary Information. The thermal stability of the synthesized epoxy microcapsules and amine microcapsules was characterized using a thermogravimetric analyzer (TG 209 F3, NETZSCH, Selb, Germany). The effective core fraction of epoxy microcapsules and amine microcapsules was measured using a physical separation method by extracting the core liquid using toluene, and the specific values were listed in Table 1.

## 3. Results and Discussion

### 3.1. Synthesis of the Dual Microcapsules

When synthesizing microcapsules by the ES-IP microencapsulation technique, the microcapsule size is mainly controlled by adjusting the spraying voltage and the core liquid feeding rate [12]. When the microcapsule size to be prepared is small, it can be achieved by increasing the spraying voltage or reducing the core liquid feeding rate. However, since the spraying voltage cannot be increased without limitation, reducing the core liquid feeding rate is a more practical option when the microcapsule size to be prepared is particularly small (such as ≤50 μm). However, reducing the feeding rate inevitably limits the preparation efficiency of the microcapsules. Therefore, to improve the preparation efficiency of small-sized microcapsules, this investigation innovatively adopts double nozzles on the basis of the traditional ES-IP microencapsulation technique.

**Figure 3:**
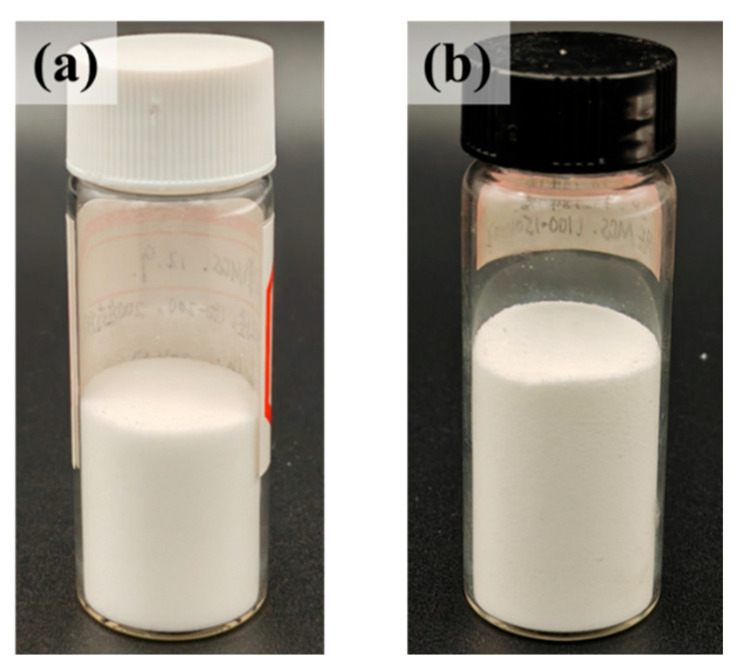
(**a**) Epoxy microcapsules and (**b**) amine microcapsules synthesized using the double-nozzle ES-IP microencapsulation technique.

Figure 3a,b show the photos of epoxy microcapsules and amine microcapsules prepared by the double-nozzle ES-IP microencapsulation technique, in which the sizes of epoxy microcapsules and amine microcapsules are 80 ± 10 μm and 75 ± 15 μm, respectively. The synthesized epoxy microcapsules and amine microcapsules possess high storage stability. The epoxy microcapsules and amine microcapsules synthesized using the ES-IP microencapsulation technique exhibit excellent storage stability. After being sealed and stored at room temperature for up to seven months, the appearance of both microcapsules remains virtually unchanged compared to their freshly synthesized states. When the sizes of the two microcapsules are larger than 50 μm, they can form dry microcapsules that can be collected, have good dispersibility, and can flow freely like sand. Although the ES-IP microencapsulation technique can still synthesize microcapsules with a particle size of 20~50 μm, the two microcapsules are prone to agglomerating and forming lumps during drying due to the size effect, and cannot be collected into dispersed and freely flowing microcapsules. Therefore, if small-sized microcapsules are used, they can be transferred to other materials in the form of a microcapsule dispersion using a phase transfer agent.

**Figure 4:**
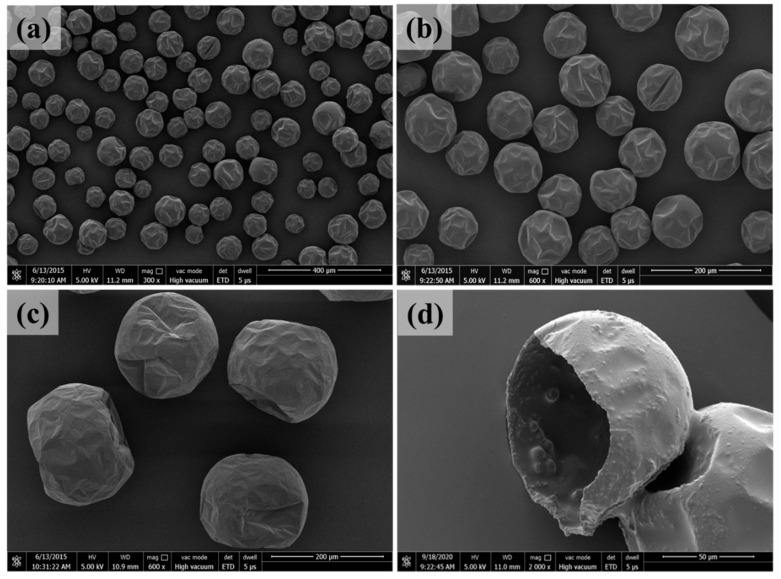
SEM images of the synthesized epoxy microcapsules with different sizes. (**a**) ~50 μm, (**b**) 50~100 μm, (**c**) 150~200 μm, and (**d**) a fractured epoxy microcapsule showing core–shell structure.

**Figure 5:**
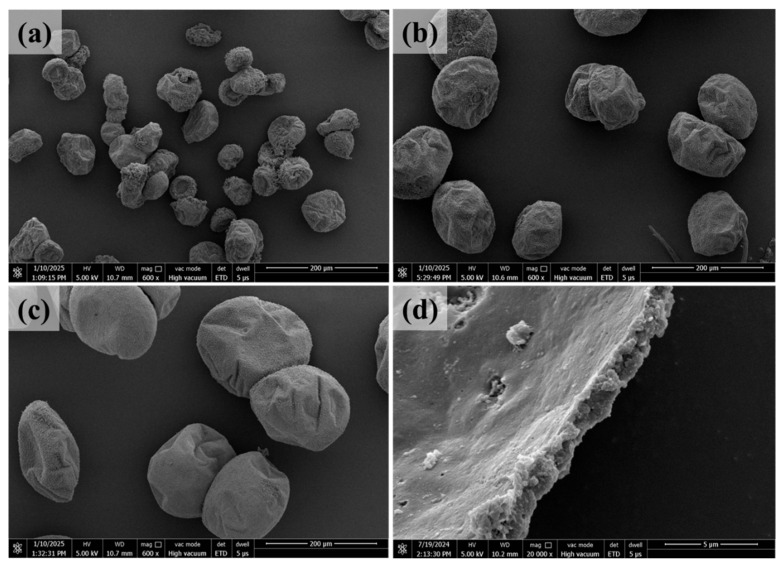
SEM images of the synthesized amine microcapsules with different sizes. (**a**) ~50 μm, (**b**) 50~100 μm, (**c**) 150~200 μm, and (**d**) a fractured amine microcapsule showing core–shell structure.

Figure 4 and Figure 5, respectively, are SEM images of epoxy microcapsules and amine microcapsules with different sizes. The particle size and distribution of microcapsules prepared by double-nozzle spraying are not significantly different from those prepared by single-nozzle spraying; that is, in the double-nozzle spraying process, the microdroplets sprayed from different nozzles do not appear to fuse to increase the microcapsule size. Since the microdroplets generated by electrostatic spraying carry the same charges, there is Coulomb repulsion among them. Therefore, even if double nozzles are used, the microdroplets sprayed from different nozzles are difficult to fuse. In addition, as can be seen from the figure, the microcapsule size can be easily controlled by adjusting the spraying voltage and the feeding rate of the core liquid. Overall, regardless of the particle size, the epoxy microcapsules have better dispersibility, and even when the particle size is as small as 50 μm, there is no adhesion between all microcapsules. In addition, under different particle sizes, the morphology of the epoxy microcapsules is relatively regular and consistent. Different from epoxy microcapsules, due to the high reactivity, strong amphiphilicity, and high solubility in the reaction solution of the adopted polyamines, it is more difficult to prepare amine microcapsules. Although the prepared amine microcapsules still have good dispersibility, their shape, dispersibility, etc., are not as good as epoxy microcapsules. Figure 4d and Figure 5d, respectively, are the cross-sections of the microcapsules after they were opened and the core liquid was removed with toluene. As shown in the figure, the microcapsule shell of the epoxy microcapsules is dense and uniform, with a thickness of about 1.2 μm; the inner wall of the amine microcapsules is thin and dense, with a thickness of about 2 μm. Overall, the microcapsule shells of both healant microcapsules can provide good protection for their core liquids, so that their core liquids can withstand the harsh curing process of the resin matrix and provide self-healing performance.

**Figure 6:**
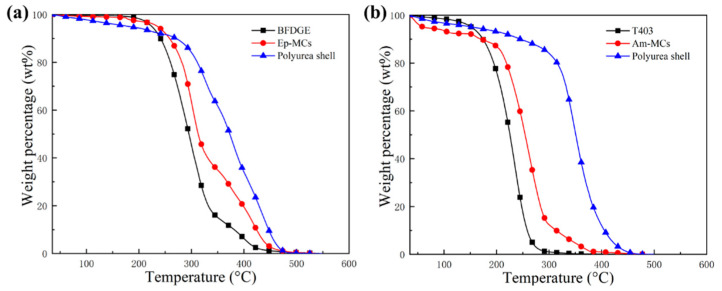
(**a**) TGA curves of the epoxy microcapsules of size in 50~100 μm, the pure epoxy healant BFDGE, and the extracted pure polyurea shell. (**b**) TGA curves of the amine microcapsules of size in 50~100 μm, the pure amine healant T403, and the extracted pure polyurea shell.

The thermal stability of the synthesized epoxy microcapsules and amine microcapsules was characterized by TGA. Figure 6a shows the TGA curves of the epoxy microcapsules of size 50~100 μm, the pure epoxy healant BFDGE, and the extracted pure polyurea shell. It shows that the epoxy microcapsule and the adopted epoxy healant BFDGE are quite stable without evident weight loss before 200 °C. Although a slight weight loss of about 7 wt% is observed for the polyurea shell, it can be considered as thermally stable before this temperature. Similarly, Figure 6b shows the TGA curves of the amine microcapsule of size in 50~100 μm, the pure amine healant T403, and the polyurea shell. A weight loss of about 5 wt% for the three specimens before 150 °C indicates that they will be relatively stable during the curing process and the service life of this self-healing material.

### 3.2. Self-Healing Performance

This investigation further systematically studied the healing performance of this self-healing imidazole-cured epoxy based on the dual microcapsules, including the effects of the ratio of the dual microcapsules, the concentration of the microcapsules, and the size of the microcapsules on the healing performance. The specific parameters selected are shown in Table 2. The healing conditions after the sample fracture were 48 h at room temperature.

#### 3.2.1. Optimization of Microcapsule Ratio

First, this investigation studied the effect of the ratio of epoxy microcapsules to amine microcapsules on the self-healing performance of imidazole-cured epoxy. The sizes of the two microcapsules were between 50~100 μm, and the total concentration was 10.0 wt%.

**Figure 7:**
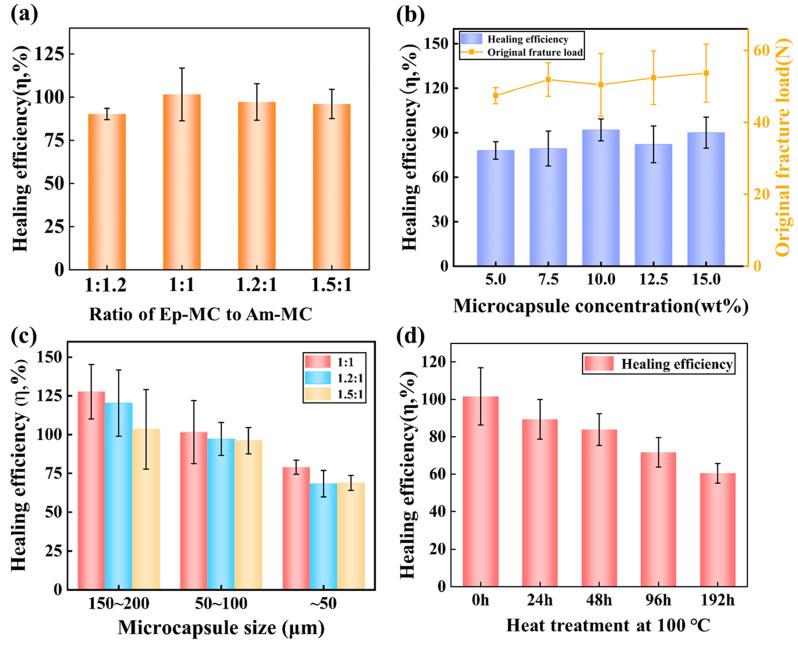
Self-healing performance of the formulated self-healing epoxy with respect to: (**a**) Ratio of epoxy microcapsule to amine microcapsule when the total microcapsule concentration is 10.0 wt%, (**b**) Total microcapsule concentration when the ratio of epoxy microcapsule to amine microcapsule is 1:1, (**c**) Microcapsule size when the total microcapsule concentration is 10.0 wt% and the ratio of the two microcapsules is 1:1, and (**d**) Heat treatment duration at 100 °C when the total microcapsule concentration is 10.0 wt% and the ratio of the two microcapsules is 1:1.

Figure 7a shows the trend of the self-healing efficiency with the ratio of the epoxy microcapsules to amine microcapsules when the ratio varies from 4.5 wt%:5.5 wt% (1:1.2) to 6.7 wt%:3.3 wt% (1.5:1). Compared with other self-healing epoxies using this microencapsulated healant system [13,16] or self-healing epoxies using other microcapsules [21,22,23], the self-healing imidazole-cured epoxy can obtain very high self-healing performance even when the microcapsule size is very small (about 75 μm) and the curing conditions are relatively harsh (60 °C—2 h + 100 °C—2 h + 120 °C—4 h + 140 °C—5 h). When the microcapsule ratio is 1:1, the optimal healing efficiency is as high as 100%. Although the amine microcapsules in the imidazole-cured epoxy will partially lose the core liquid during the high-temperature curing process [24], the above results show that the curing process did not have a significant impact on the healing performance of this self-healing material. In addition, the healing performance of the self-healing material is insensitive to the ratio variation in the two microcapsules. In a wide range of 4.5:5.5 to 6.7:3.3, the healing efficiency of all samples is higher than 90%. Considering that the microcapsules in the microcapsule-based self-healing material are discretely randomly distributed, and the agglomeration phenomenon of microcapsules becomes more and more serious as the particle size decreases, it is of high practical significance that the self-healing efficiency of the two-part self-healing system is insensitive to the microcapsule ratio in a wide range of microcapsule ratios.

The reason why the self-healing efficiency of this microcapsule-based self-healing system is high is that on the one hand, the healant system is homogeneous with the matrix; that is, epoxy-related healants are used to heal the epoxy matrix. Owing to this homogeneity, the high healability of the healant system to the imidazole-cured epoxy matrix is still well preserved after being microencapsulated. On the other hand, the brittleness of the imidazole-cured epoxy matrix is high, and the curing product of the selected healant system has good toughness. Therefore, it is also reasonable that the self-healing imidazole-cured epoxy resin based on the microencapsulated healant system has high self-healing efficiency.

#### 3.2.2. Effect of Microcapsule Concentration

On the basis of optimizing the microcapsule ratio, this investigation studies the effect of the microcapsule concentration in the self-healing epoxy on the healing performance. Figure 7b shows the trend of the initial fracture load and self-healing efficiency of the samples when the ratio of the two microcapsules is 1:1, the microcapsule size is 50~100 μm, and the concentration changes from 5.0 wt% to 15.0 wt%. Similarly to other microcapsule-based self-healing materials [23,25,26], microcapsules have a certain toughening effect on the matrix material, but the toughening effect is not as significant as of others. Overall, the self-healing efficiency of the self-healing imidazole-cured epoxy tends to increase with the increase in the microcapsule concentration, but the improvement effect is not very significant. On the contrary, the self-healing performance of the self-healing system at low microcapsule concentration is more eye-catching, and the self-healing efficiency at a concentration of 5.0 wt% is as high as 78.1 ± 5.9%. Since the imidazole-cured epoxy resin used in this investigation will eventually be used as the matrix of fiber-reinforced resin-based composite materials, the space for the resin and microcapsules inside the composite material is limited. Therefore, it is more appropriate to obtain high self-healing efficiency at low microcapsule concentration.

#### 3.2.3. Effect of Microcapsule Size

On the basis of optimizing the microcapsule ratio and concentration, this investigation further studied the effect of microcapsule size on the healing performance of the self-healing imidazole-cured epoxy. Figure 7c shows the trend of the self-healing efficiency of the system with the particle size when the ratio of epoxy microcapsules to amine microcapsules is 1:1, 1.2:1, and 1.5:1, respectively. As can be seen from the figure, when the microcapsule size is large (150~200 μm), regardless of the ratio of the two microcapsules, the healing efficiency of the self-healing material exceeds 100%, and the highest exceeds 125%. However, as the microcapsule size decreases, its healing efficiency decreases accordingly. When the microcapsule size is as small as about 50 μm, the healing efficiency is about 75%. It can be seen that although it has experienced a relatively harsh curing process, its healing efficiency is still at a high level. The influence of microcapsule size on the self-healing imidazole-cured epoxy is consistent with that of other microcapsule-based self-healing materials; that is, under the same other conditions, the smaller the microcapsule size, the smaller the dose of healant that can be released to the fracture surface, so the self-healing performance decreases [27,28]. Considering that the self-healing imidazole-cured epoxy will be used as the matrix of fiber-reinforced resin-based composite materials in the future, and the limited space between the fiber layers will greatly affect the survival performance of large-sized microcapsules, it is of great significance that high self-healing performance can be achieved when the microcapsule size is small, for further developing self-healing fiber-reinforced resin-based materials based on the resin system.

#### 3.2.4. Effect of Heat Treatment Conditions

Since materials often work under heating conditions, it is of high practical importance to study the thermal stability of the healing performance of the self-healing material. This investigation placed the optimized samples at a relatively high temperature (such as 100 °C) for a certain duration, and then characterized their self-healing performance. Figure 7d shows the trend of the healing efficiency of the self-healing imidazole-cured epoxy containing 10.0 wt% 50~100 μm microcapsules with the heat treatment duration. As the heat treatment duration is prolonged, the healing efficiency gradually decreases. However, it still retains a healing efficiency of up to 60% even after being treated at 100 °C for 192 h (i.e., 8 days). For polymeric materials, 100 °C is already a high-use temperature. Therefore, the above experimental results can show that the self-healing imidazole-cured epoxy has high thermal stability.

#### 3.2.5. Fractography

**Figure 8:**
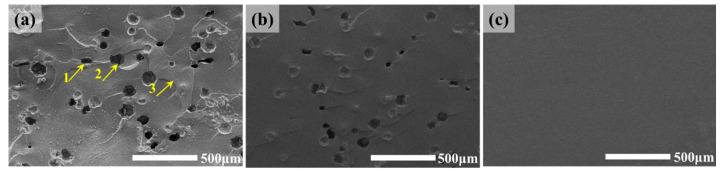
Fractography of (**a**) self-healing epoxy, (**b**) self-healing epoxy with removal of the released healants after fracture, and (**c**) pure epoxy.

In this investigation, the fracture surface of the self-healing imidazole-cured epoxy containing 5.0 wt% 50~100 μm microcapsules (epoxy microcapsule/amine microcapsule = 1:1) after healing was analyzed (Figure 8a), and compared with the self-healing imidazole-cured epoxy that was broken but not healed section (Figure 8b, the fracture surface was thoroughly rinsed using toluene to remove the released healants) and the pure imidazole-cured epoxy without microcapsules (Figure 8c).

Arrows 1 and 2 in Figure 8a indicate a broken epoxy microcapsule and a broken amine microcapsule on the fracture surface, respectively. The big cavities still present in the microcapsules after rupture indicate that the microcapsule shell provides good sealing, and the epoxy adhesive outside the microcapsules does not enter the microcapsules during the resin curing process. Compared with the pure epoxy fracture (Figure 8c), the fracture surface of the epoxy containing microcapsules (Figure 8a,b) is obviously rougher, showing the toughening effect of the microcapsules on the epoxy matrix [25]. Compared with the fracture surface that was thoroughly removed, the healants released by the microcapsules with toluene (Figure 8c), some substance appeared on the fracture surface of the self-healing epoxy, as shown by arrow 3. This substance is the solidified product of the healant released by the microcapsules to achieve in situ bonding of the crack planes, that is, self-healing. Since the small-sized microcapsules used carry less healant than the large-sized microcapsules and lose a part of the healant during the curing process of the epoxy matrix, only discontinuous solidified products of the healant can be observed on the fracture surface. This result is also consistent with the healing efficiency based on artificially injecting a sufficient amount of premixed healant. If the fracture surface is completely filled with the healant, like the latter, the self-healing efficiency will be as high as 200%. Nevertheless, due to the high efficiency of the selected healant system, the self-healing epoxy can still obtain a healing efficiency of up to 100% even when the healant can only discontinuously fill the crack.

### 3.3. Mechanical Properties of the Self-Healing Epoxy

**Figure 9:**
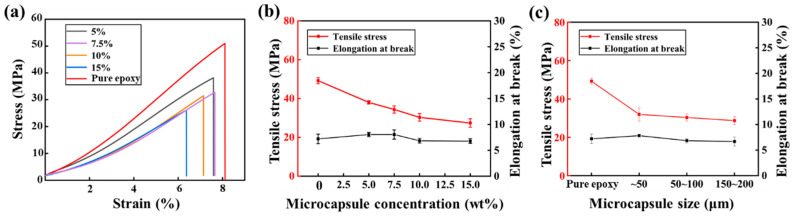
(**a**) Original stress–strain curves of the self-healing epoxies with different microcapsule concentrations, (**b**) Tensile stress and elongation at break of the self-healing epoxies with respect to microcapsule concentration, and (**c**) Tensile stress and elongation at break of the self-healing epoxies with respect to microcapsule size.

In addition to toughening, this investigation studies the effect of the added microcapsules on the tensile properties of the self-healing imidazole-cured epoxy. Figure 9a,b show the typical original stress–strain curves, tensile strength, and elongation at break of the self-healing imidazole-cured epoxy with the increase in microcapsule concentration, respectively. As can be seen from the figure, the added microcapsules reduce the tensile properties of the self-healing material. When the microcapsule concentration is 5.0 wt%, the tensile strength of the imidazole-cured epoxy decreases from 49.2 ± 1.6 MPa of the pure resin to 38.0 ± 0.8 MPa, a decrease of about 22%. When the microcapsule concentration further increases, the strength of the self-healing material decreases accordingly. At the optimal self-healing performance (microcapsule concentration 10.0 wt%), the strength is 30.3 ± 1.9 MPa, a decrease of about 38% compared with the pure resin. The decrease in the tensile properties of the self-healing material is consistent with that of other microcapsule-based self-healing materials [26,27]. This is mainly attributed to the fact that the microcapsules containing huge liquid-core cavities are equivalent to spherical defects in the matrix [12,16,26,27]. Compared with the significant decrease in tensile strength, the elongation at break of the self-healing material does not show obvious changes, and generally remains at about 8%. The main reason for this phenomenon is that the imidazole-cured epoxy itself is brittle, and the added microcapsules have a certain toughening effect.

While keeping the total microcapsule concentration at 10.0 wt%, this investigation further studies the effect of microcapsule size on the tensile properties of the self-healing material, and the results are shown in Figure 9c. Regardless of the microcapsule size, the tensile strength decreased significantly once added to the matrix, while the elongation at break remained essentially unchanged compared to the pure resin. As the microcapsule size increases, the tensile strength of the self-healing material exhibited a slight decrease. Although at the same concentration, the number of microcapsules in the matrix is higher when the microcapsule size is smaller compared to when the size is larger, the stress concentration caused by larger microcapsules during tensile testing is more significant, leading to more severe degradation of the material’s tensile properties. Therefore, the decrease in tensile strength is more pronounced when the microcapsule size is larger.

**Figure 10:**
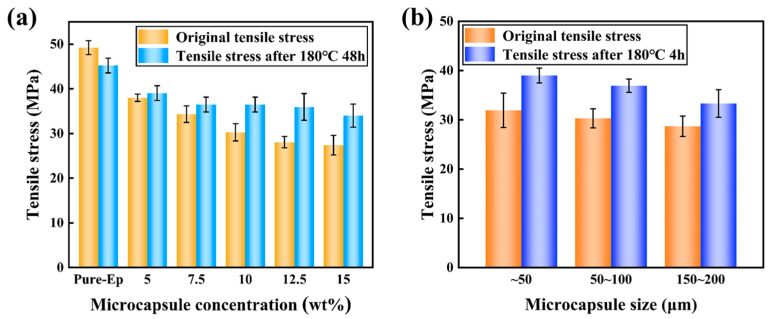
Tensile stress of the self-healing epoxy after heat treatment at 180 °C for 48 h. (**a**) Influence of the microcapsule concentration, and (**b**) Influence of the microcapsule size.

Furthermore, considering that the imidazole-cured epoxy used in this study may be subjected to high-temperature thermal shock during service, we also investigated the effect of thermal shock after adding microcapsules on the tensile properties of the material. Figure 10a,b show the changes in tensile strength of the self-healing material with different microcapsule concentrations and sizes before and after thermal shock at 180 °C for 48 h. As can be seen from the figures, for the pure imidazole-cured epoxy, the tensile strength decreased significantly after thermal shock at 180 °C for 48 h, decreasing from 49.2 ± 1.5 MPa to 45.2 ± 1.7 MPa, a reduction of approximately 8%. The main reason for this performance degradation is that the material underwent aging under high-temperature thermal shock, leading to a decrease in mechanical properties. However, the tensile strength of the self-healing imidazole-cured epoxy after thermal shock at 180 °C for 48 h was higher than that before thermal shock, and the higher the microcapsule concentration, the more significant the increase in tensile strength. In addition, the strength change in the self-healing material with different microcapsule sizes also had a similar effect, as shown in Figure 10b. It indicates that adding microcapsules can significantly improve the resistance of the self-healing imidazole-cured epoxy to thermal shock. The reason for this phenomenon is that the healants in the microcapsules, namely epoxy monomers and amine curing agents, diffuse into the matrix during thermal shock. The healing agents remaining in the matrix have a certain plasticizing effect on the matrix, improving the crack sensitivity of the brittle material and enhancing its tensile properties. The appearance of this thermal shock strengthening effect has important practical significance for the potential applications of the self-healing material.

## 4. Conclusions

This study investigates the feasibility of developing self-healing in epoxy materials for reactors, specifically imidazole-cured epoxy resins, based on microencapsulated epoxy-amine chemistry. It explores the impact of microcapsule addition on the self-healing and mechanical properties of the imidazole-cured epoxy. Initially, this paper attempts to prepare epoxy microcapsules and amine microcapsules using the ES-IP microencapsulation technique with a double-nozzle setup. Due to the electrostatic repulsion between microdroplets generated by electrospraying, these microdroplets do not coalesce. Consequently, the microcapsule size and distribution prepared using the double-nozzle ES-IP microencapsulation technique are basically consistent with those prepared using the single-nozzle technique. The two synthesized microcapsules exhibit excellent dispersibility, controllable and adjustable particle sizes ranging from 20 μm to several hundred microns, good shell tightness, and high core content. Based on these synthesized high-performance epoxy microcapsules and amine microcapsules, this paper investigates the influence of the ratio, concentration, and size of the two microcapsules on the healing performance of the self-healing imidazole-cured epoxy. The results show that the self-healing epoxy containing 10.0 wt% 50~100 μm microcapsules achieves a maximum healing efficiency of approximately 100% when the ratio of the epoxy microcapsule to amine microcapsule is 1:1. Furthermore, this self-healing epoxy is insensitive to the microcapsule ratio, maintaining a self-healing efficiency of up to 90% within a wide range of microcapsule ratios. The healing efficiency of the self-healing epoxy increases with increasing microcapsule concentration, and even at a low concentration of 5.0 wt%, the self-healing efficiency is as high as 80%. Microcapsule size has a significant impact on the healing performance of this self-healing imidazole-cured epoxy. When the microcapsule size is 150~200 μm, the self-healing efficiency reaches approximately 130%. As the microcapsule size decreases, the healing performance decreases accordingly. However, even when the microcapsule size is only approximately 50 μm, the self-healing efficiency of this system can reach approximately 80%. The self-healing imidazole-cured epoxy also exhibits high thermal stability. During heat treatment at 100 °C, although the self-healing efficiency decreases to some extent as the treatment time increases, this self-healing material still retains approximately 60% healing efficiency even after a treatment time of up to 192 h. Finally, this paper studies the effect of microcapsule addition on the mechanical properties of the material. It shows that although microcapsules have a certain toughening effect on imidazole-cured epoxy, they reduce its tensile strength. However, the added microcapsules significantly increase the material’s resistance to high-temperature aging. Due to the toughening effect of the healant diffusing from the microcapsules into the matrix at high temperatures, the tensile strength of the self-healing samples treated at 180 °C for 48 h is higher than that of the untreated ones, compared to the significant decrease in the mechanical properties of pure imidazole-cured epoxy after high-temperature aging. The above research lays a solid foundation for the development of self-healing reactor composite encapsulations based on microencapsulated epoxy-amine chemistry.

## Figures and Tables

**Table 1:** Specific feeding rates and applied electrospraying voltages for the fabrication of epoxy microcapsules and amine microcapsules with different sizes.

Microcapsule Types	Diameter (μm)		Feeding Rate ^a^ (mL/h)	Spraying Voltage (kV)	Microencapsulation Efficiency (g/h)	Core Frarction (wt%)
Epoxy microcapsule	~50	52 ± 5	2	18	3.28	80.3
	50~100	80 ± 10	5	20	8.70	83.5
	150~200	180 ± 8	20	22	37.20	88.6
Amine microcapsule	~50	50 ± 8	3	28	3.01	75.7
	50~100	75 ± 15	8	20	12.96	78.4
	150~200	178 ± 10	20	15	35.20	84.1

^a^ The feeding rate of one nozzle.

**Table 2:** Specific parameter combinations for the self-healing epoxy adopted in this study.

#	Mass Ratio (Ep-MC: Am-MC)	Microcapsule Diameter (μm)	Microcapsule Content (wt%)	Post-Heat Treatment (h)
1	1:1.2	50~100	10.0	-
2	1:1	50~100	10.0	-
3	1.2:1	50~100	10.0	-
4	1.5:1	50~100	10.0	-
5	1:1	~50, 50~100, 150~200	5.0	-
6	1:1	~50, 50~100, 150~200	7.5	-
7	1:1	~50, 50~100, 150~200	10.0	-
8	1:1	~50, 50~100, 150~200	12.5	-
9	1:1	~50, 50~100, 150~200	15.0	-
10	1:1	50~100	10.0	0
11	1:1	50~100	10.0	24
12	1:1	50~100	10.0	48
13	1:1	50~100	10.0	96
14	1:1	50~100	10.0	192

**Table 3:** Specific parameter combinations of the samples for tensile test.

#	Mass Ratio (Ep-MC: Am-MC)	Microcapsule Diameter (μm)	Microcapsule Content (wt%)	Post-Heat Treatment
1	1:1	50~100	5.0	-
2	1:1	50~100	7.5	-
3	1:1	50~100	10.0	-
4	1:1	50~100	12.5	-
5	1:1	50~100	15.0	-
6	1:1	~50	10.0	-
7	1:1	50~100	10.0	-
8	1:1	150~200	10.0	-
9	1:1	50~100	10.0	180 °C—48 h

## Data Availability

Data are contained within the article and Supplementary Materials.

## Supplementary Materials

The following supporting information can be downloaded at: https://www.mdpi.com/article/10.3390/polym17172391/s1, Figure S1: Size distribution of the synthesized microcapsules of 50~100 μm. (a) Epoxy microcapsules. (b) Amine microcapsules.
