# Supplementary material for: Self-Healing Imidazole-Cured Epoxy Using Microencapsulated Epoxy-Amine Chemistry
Source: Polymers (Basel). 2025 Sep 1;17(17):2391. doi: 10.3390/polym17172391 (PMC12431301; doi:10.3390/polym17172391)
Supplement: Supplementary file 1 [file polymers-17-02391-s001.zip › polymers-3826066-supplementary.pdf]

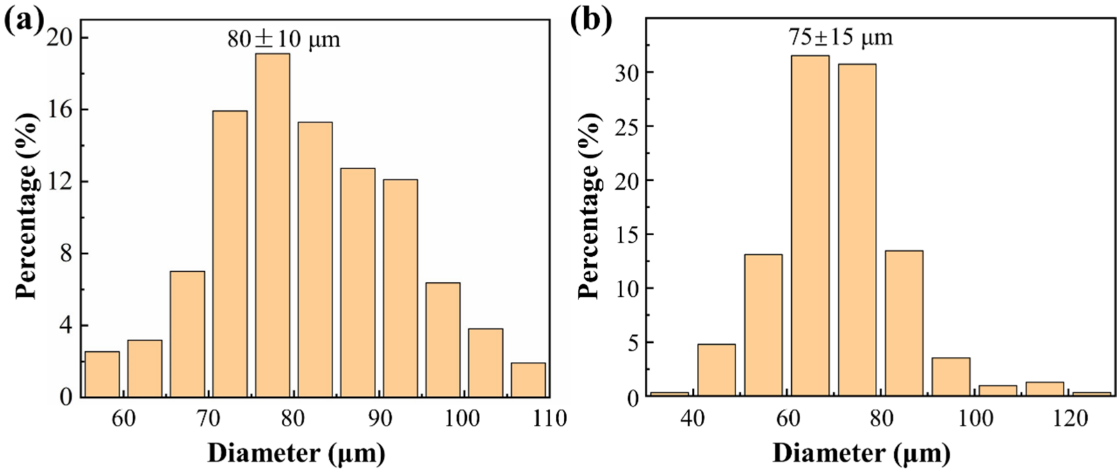

Figure S1. Size distribution of the synthesized microcapsules of 50~100 μm. (a) Epoxy microcapsules. (b) Amine microcapsules.
